# Root exudation patterns of contrasting rice (*Oryza sativa* L.) lines in response to P limitation

**DOI:** 10.1007/s00425-024-04556-2

**Published:** 2024-10-23

**Authors:** Henning Schwalm, Christiana Staudinger, Mohammad-Reza Hajirezaei, Eva Mundschenk, Alireza Golestanifard, Maire Holz, Matthias Wissuwa, Eva Oburger

**Affiliations:** 1 Department of Forest and Soil Science, Institute of Soil Research, University of Natural Resources and Life Sciences, 3430 Tulln an der Donau, Vienna Austria; 2 Department of Crop Sciences, Institute of Soil Research, University of Natural Resources and Life Sciences, 3430 Tulln an der Donau, Vienna Austria; 3 Department of Physiology and Cell Biology, Leibniz Institute of Plant Genetics and Crop Plant Research, Gartersleben, Germany; 4 Group of Isotope Biogeochemistry and Gas Fluxes, Leibniz Centre for Agricultural Landscape Research (ZALF) e.V., Müncheberg, Germany; 5 Crop, Livestock and Environment Division, Japan International Research Center for Agricultural Sciences, Tsukuba, Japan

**Keywords:** Amino acids, Carbohydrates, Carboxylates, Phenolics, Phosphorus

## Abstract

**Main conclusion:**

Rice exudation patterns changed in response to P deficiency. Higher exudation rates were associated with lower biomass production. Total carboxylate exudation rates mostly decreased under P-limiting conditions.

**Abstract:**

Within the rhizosphere, root exudates are believed to play an important role in plant phosphorus (P) acquisition. This could be particularly beneficial in upland rice production where P is often limited. However, knowledge gaps remain on how P deficiency shapes quality and quantity of root exudation in upland rice genotypes. We therefore investigated growth, plant P uptake, and root exudation patterns of two rice genotypes differing in P efficiency in semi-hydroponics at two P levels (low P = 1 µM, adequate P = 100 µM). Root exudates were collected hydroponically 28 and 40 days after germination to analyze total carbon (C), carbohydrates, amino acids, phenolic compounds spectrophotometrically and carboxylates using a targeted LC–MS approach. Despite their reported role in P solubilization, we observed that carboxylate exudation rates per unit root surface area were not increased under P deficiency. In contrast, exudation rates of total C, carbohydrates, amino acids and phenolics were mostly enhanced in response to low P supply. Overall, higher exudation rates were associated with lower biomass production in the P-inefficient genotype Nerica4, whereas the larger root system with lower C investment (per unit root surface area) in root exudates of the P-efficient DJ123 allowed for better plant growth under P deficiency. Our results reveal new insights into genotype-specific resource allocation in rice under P-limiting conditions that warrant follow-up research including more genotypes.

**Supplementary Information:**

The online version contains supplementary material available at 10.1007/s00425-024-04556-2.

## Introduction

Phosphorus (P) is often a major growth-limiting factor in agroecosystems, particularly on highly weathered tropical soils where upland rice is typically cultivated (Lynch 2011; Fageria and Oliveira 2014; Saito et al. 2019). As essential plant macronutrient, P is a constituent of various organic molecules (e.g., nucleic acids, phospholipids) and involved in metabolic energy transfer processes in the form of ATP, explaining its vital role in photosynthesis and respiration, and subsequently in plant growth (Plaxton and Tran 2011). An improved understanding of and consequent breeding for root and rhizosphere traits involved in efficient P acquisition is considered a key strategy for rendering agricultural production more sustainable (Bishopp and Lynch 2015; Oburger et al. 2022a, b). Plants increase P acquisition by (i) enhanced top soil exploration via increased lateral root growth (Lynch and Brown 2001) and/or in association with mycorrhizal fungi (Smith et al. 2003), as well as via (ii) increased soil mining driven by enhanced exudation of organic compounds (Lambers et al. 2006) and by nurturing beneficial rhizosphere-associated microbes (Richardson and Simpson 2011). While the importance of enhanced soil exploration has been repeatedly demonstrated (Lynch and Brown 2001; Wissuwa et al. 2020), the role of root exudation in improving P acquisition in rice is still not well understood.

Root exudates are plant metabolites released by roots into the surrounding soil. They are key components within the rhizosphere as they mediate the interaction between plant roots, the soil matrix and the root-associated microbiome (Vives-Peris et al. 2019). Various low molecular weight root exudates were found to increase nutrient desorption from soil minerals and organic matter which renders the nutrients more available for plant uptake (Clarholm et al. 2015; Chai and Schachtman 2022). However, the relationship between root exudation and nutrient availability is bidirectional. Root exudates are synthesized from photosynthates and the very same nutrients that exudate help acquire simultaneously function as important building blocks and regulatory molecules in plant metabolism. Consequently, root exudates not only modify nutrient availability in the rhizosphere but also the overall plant nutrient status, which in turn can affect root exudation (Carvalhais et al. 2011; Canarini et al. 2019).

Differences in the ability to maintain sufficient growth and yield under nutrient limiting conditions have been repeatedly reported among various crop genotypes (Sadana et al. 2002; Paponov et al. 2005; Wacker-Fester et al. 2019). The difference in growth between genotypes grown under a certain concentration of P supply (here defined as P efficiency, George et al. 2011) is determined by differences in internal P use efficiency, as well as different belowground P acquisition strategies (root exploration, mining). However, disentangling responsible traits and mechanisms is challenging. In rice, one of the worlds’ most important staple food crops, the P-efficient genotype DJ123 has been shown to produce higher yields in soils with low P availability, compared to less P-efficient genotypes, such as Nerica4 (Wissuwa et al. 2020). Focusing on belowground traits, differences in root morphological traits between these genotypes partially explained the superior P acquisition of DJ123 (Wissuwa et al. 2020), but exudate-induced changes in the rhizosphere were hypothesized to additionally affect changes in P efficiency between the two rice varieties (Mori et al. 2016).

Previous studies showed that root exudation rates of specific carbohydrates, amino acids, organic acids and phenolics can increase in maize (*Zea mays*) and bean (*Phaseolus vulgaris*) under P limitation (Juszczuk et al. 2004; Carvalhais et al. 2011). Metal complexing root exudates have the potential to contribute to P availability by triggering iron-/ aluminum-oxide dissolution via surface complexation, thereby co-liberating surface bound P or by directly mobilizing adsorbed P via ligand exchange. In particular, the anions of di- and tri-carboxylic organic acids, such as citrate, malate or oxalate (hereinafter summarized as carboxylates), are known to mobilize P and thereby improve P acquisition (Hoffland et al. 2006; Oburger et al. 2011; Ding et al. 2021). Uncharged molecules, such as most carbohydrates, or charged molecules with low metal complexation affinity, such as proteinogenic amino acids, are unlikely to solubilize P directly, but can stimulate microbial activity and turnover in the rhizosphere and thereby contribute to plant nutrient uptake (Carvalhais et al. 2011; Raymond et al. 2021). In rice, enhanced carboxylate exudation under P deficient conditions has been reported (Hoffland et al. 2006), but the effect of P deficiency on exudation rates of other organic compounds which may be important for microbial community assembly has not been extensively studied.

Understanding the response of root exudation to P limitation is a prerequisite to unravel the potential role of exudation in improved P acquisition of upland rice. We therefore investigated the effect of P deficiency on exudation of total organic C and other main exudate compound classes including carbohydrates, amino acids, phenolics and carboxylates, at two time points [i.e., 28 and 40 days after germination (DAG)] in two rice genotypes with contrasting P efficiencies (DJ123 and Nerica4) in a semi-hydroponic setup with limiting and adequate P supply. We hypothesized that the exudation rates of total C and of the different exudate compound classes investigated (carbohydrates, amino acids, phenolics and carboxylates) increase in response to P limitation (H1), and are greater in the P-efficient genotype DJ123 compared to inefficient Nerica4 (H2), and that exudation rates decrease over time (H3).

## Materials and methods

### Plant material and experimental design

Two contrasting upland rice (*Oryza sativa* L.) genotypes were used in this study: DJ123 with high P efficiency and Nerica4 with low P efficiency (Wissuwa et al. 2020). Seeds were surface-sterilized in 15% NaOCl for 3 min and washed prior to germination on filter paper imbibed with 12 μM FeEDTA and 0.1 mM CaCl_2_ at room temperature for seven days. Germinated rice seedlings were transferred into free draining 2 L pots filled with 2.8 kg of quartz sand (Quarzwerke Österreich GmBH, Melk, Austria) and with half strength Yoshida solution (Yoshida et al. 1972) with an adjusted pH of 6 prepared in deionized water with P concentrations of either 1 µM (low P) or 100 µM (adequate P supply). The semi-hydroponic experiment was conducted in a greenhouse with day/night temperatures of 30 °C/24 °C, air humidity of 70%, and a photosynthetic photon-flux density of min. 396 µmol m^−2^ s^−1^ (PAR). Six replicates per genotype and per P treatment were grown in a completely randomized 2 × 2 factorial design. Plants reached average phenological growth stages of BBCH 14 (four leaves unfolded) and BBCH 16 (six leaves unfolded) at 28 and 40 DAG, respectively (Bleiholder et al. 2001).

### Root exudate sampling

At 28 and 40 DAG, root exudates were collected according to the soil-hydroponic-hybrid approach (Oburger et al. 2014; Santangeli et al. 2024). High-quality (HQ) water (conductivity 0.055–0.08 μS cm^−1^; Thermo Electron LED GmbH, Niederelbert, Germany) was used to prepare an exudate sampling solution containing 10 mg L^−1^ sterilizing agent (Micropur® Classic MC 1’000F; Katadyn Group, Kemptthal, Switzerland). Briefly, roots were gently rinsed with deionized water to remove the sand and clean root systems of intact plants were transferred to containers containing fresh solution (later to be discarded) for osmotic adjustment for 5 min. Thereafter, plants were placed into opaque containers containing 0.3 L (28 DAG) or 0.5 L (40 DAG) of exudate sampling solution. Sampling containers with submerged roots were wrapped in aluminum foil and moved back into the greenhouse for 3 h of exudate collection. Exudate solution was then filtered with 0.2 μm cellulose acetate filters (Chromafil™ CA-20/25 (S); Macherey–Nagel™ GmbH, Düren, Germany) aliquoted and frozen at −20 °C. Aliquots were freeze-dried and reconstituted in a smaller volume with HQ water prior to spectrophotometric and LC–MS analyses.

### Spectrophotometric analysis of total C, carbohydrates, amino acids and phenolic compounds

A spectrophotometry-based assay was conducted to quantify dissolved organic C (= total C) concentration in rice exudate samples (Oburger et al. 2022a, b). Absorbance of filtered original exudate solution was determined at 260 nm (Infinite 200 Pro; Tecan, Grödig, Austria) and potassium hydrogen phthalate (KHP) was used as a standard (calibration range from 1 to 49 µM). Spectrophotometric quantification of other exudate compound classes required adapted re-suspension of lyophilized exudate samples in HQ water. Applied protocols included an anthrone colorimetric assay for the determination of total carbohydrates with absorbance measured at 625 nm (Hansen and Møller 1975), a spectrofluorometric assay without ammonium correction (as ammonium concentrations were below detection limit) for amino acids with an emission wavelength of 450 nm and an excitation wavelength of 340 nm (Jones et al. 2002), and a modified Folin–Ciocalteu method for the quantification of total phenolics with absorbance measured at 765 nm (Ainsworth and Gillespie 2007). Corresponding standards for calibration were D-glucose for total carbohydrates (calibration range 0.025 to 1 mM), glycine for amino acids (calibration range 0.5 to 50 µM), and chlorogenic acid for phenolics (calibration range 5.6 to 282 µM).

### Carboxylates analysis

The concentration of carboxylates in rice exudates was assessed on an Agilent 1290 ultra-performance liquid chromatography (UPLC) system coupled to an Agilent 6490 triple quadrupole mass spectrometer (TQMS) (Agilent Technologies). Lyophilized exudate samples were re-suspended in 0.1 mL of HQ water prior to analysis. The chromatographic separation was performed on an Acquity UPLC column, HSS T3, 2.1 × 100 mm, 1.8 µm column at a flow rate of 0.6 mL min^−1^ and a column temperature of 40 °C (Waters GmbH). The separation was done with a linear gradient of solvent A (water) containing 0.5% formic acid (v/v). The run was performed in linear mode within 4.5 min. The ESI–MS/MS analysis was performed in negative ionization mode with nitrogen as drying and nebulising gas. The gas flow was set at 12 L min^−1^ at 250 °C and the nebulizer pressure was 30 psi. The capillary voltage was 2 kV and the dwell time set to 5 ms. The collision energy ranged from 9 to 69 eV depending on the masses estimated using the MassHunter optimizer software with MS2 Selected Ion Monitoring (SIM). For quantification, a calibration curve was generated from a range of 0.1 to 100 µM per mL for each metabolite. Standard mixtures were measured three times at the beginning, middle and end of sample batch to monitor the stability of the mass spectrometer. In addition, blanks of ultrapure methanol were injected after every 5 samples to avoid cross-contamination. MassHunter software (version 10.1, Agilent Technologies) was used for data acquisition and final qualitative and quantitative analysis.

### Root morphological and plant nutrient analysis

An aliquot of the intact root system was weighed and stored in 30% EtOH until root morphological analysis. The remaining root aliquot and the shoot were dried for 72 h at 60 °C to determine root and shoot biomass dry weights. Rice seeds, dried shoot and dried root aliquot were milled and digested with 65% HNO_3_ and 30% H_2_O_2_ using a microwave digestion system (MARS 6; CEM GmbH, Kamp-Lintfort, Germany) and lucerne as certified plant reference (IPE 152; LGC Standards Ltd.) to assess P concentrations with optical emission spectroscopy (ICP-OES OptimaTM 8300; Perkin-Elmer). For each genotype, plant P uptake was calculated as the amount of P present in the plant body, minus 75% of the amount of P contained in seeds. Intact root aliquots stored in EtOH were scanned (Epson Perfection V850 Pro; Seiko Epson Corporation) using the following settings: “professional mode”, 720 dpi, 8-bit grayscale, and positive film. Root scans were analyzed with WinRHIZO™ (Version 2019a; Regent Instruments Inc.). Total root length (m) and root surface area (cm^2^) were calculated by extrapolating the values of the scanned aliquot to the whole root system. Root morphology results are shown in Fig. S1 (supplementary information).

### Statistical analysis

Two-way analysis of variance (ANOVA) and Fisher’s least significant difference (LSD) test with Benjamini–Hochberg correction to account for multiple comparisons and a Permutational Multivariate Analysis of Variance (PERMANOVA) were carried out with R (version 4.4.0; The R foundation for statistical computing). R was also used to create the figures shown in this work. Data were log- or square root-transformed, if required, to approximate normality and homoscedasticity assumptions. Additionally, two-sample unequal variance t-test for pairwise comparison between time points, between P treatments and between genotype was applied (Tables S1, S2, S4). Means were considered significantly different when the (corrected) *P*-value was lower than 0.05.

## Results

### Biomass, total P uptake and shoot P concentration

**Fig. 1:**
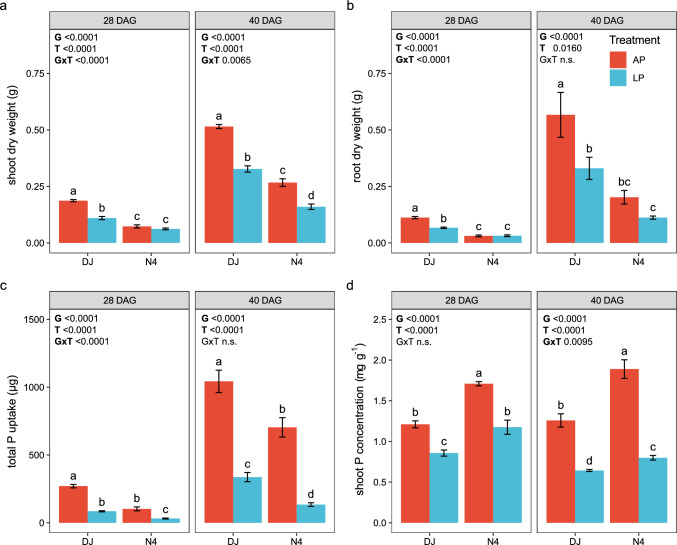
Shoot dry weight (**a**), root dry weight (**b**), total P uptake (**c**) and shoot P concentration (**d**) of rice genotypes (G) DJ123 (DJ) and Nerica4 (N4) in response to different P treatments (T) including low (LP) and adequate (AP) phosphorus supply at 28 and 40 days after germination (DAG). Values represent means ± SE, *n* = 5–6. Different letters indicate significant differences within each time point (two-way ANOVA and LSD, *P* < 0.05)

Both genotype and P supply had a significant effect on shoot and root biomass (Fig. 1a, b). Irrespective of P supply and time point, shoot and root biomass production was higher in DJ123 relative to P-inefficient Nerica4. At 28 and 40 DAG, DJ123 produced 2.9-fold and 1.9-fold more shoot biomass (4.2-fold and 3.1-fold more root biomass) than Nerica4 when P supply was adequate. Under P deficiency, shoot biomass production of DJ123 was 1.8-fold and 1.9-fold higher, while root biomass was 2.1-fold and 2.8-fold higher relative to Nerica4. Larger biomass of DJ123 was accompanied with larger root surface area and longer roots (total root length) relative to Nerica4 (Fig. S1). P deficiency treatment reduced biomass production of both rice genotypes. At 28 DAG and 40 DAG, shoot biomass of genotype DJ123 was 69% and 58% lower, while root biomass was 67% and 72% lower when grown under P deficiency (LP) relative to adequate P supply (AP) (Fig. 1a, b). Shoot and root biomass of inefficient Nerica4 did not differ between P treatments at 28 DAG, but were lower (52% less shoot, 70% less root biomass) at LP relative to AP at 40 DAG.

Total plant P uptake and shoot P concentration were also significantly reduced under P deficiency for both genotypes and harvesting dates (Fig. 1c, d). Total plant P uptake was generally higher, while shoot P concentration was lower in P-efficient DJ123 compared to inefficient Nerica4, irrespective of P supply and time point. Under P deficiency and after 28 DAG, total P uptake was 2.7-fold higher in DJ123 relative to Nerica4 (85.01 ± 3.9 µg P in DJ123 and 31.8 ± 3.4 µg P in Nerica4, mean ± SE). The genotypic difference for total P uptake decreased over time and was lower at 40 DAG (2.2-fold higher in DJ123 relative to Nerica4 at LP). In contrast, shoot P concentration was higher in Nerica4 (at LP 1.4-fold and 1.3-fold at 28 and 40 DAG, respectively) compared to DJ123 (Fig. 1d).

### Effect of time, genotype and P treatment on investigated exudate parameters

Overall, root exudation rates per root surface area of total dissolved organic C, carbohydrates, amino acids, phenolics and carboxylates were significantly affected by time, genotype and P treatment, with time as most significant factor accounting for 50% of the variation in exudation (*R*^2^ = 0.50), followed by genotype (*R*^2^ = 0.26), and P supply (*R*^2^ = 0.05) only being of minor relevance (Table 1).

**Table 1:** Permutational Multivariate Analysis of Variance (PERMANOVA) results showing the effect of factors (phosphorus treatment, genotype, time point) on root exudation rates

	*R*^2^	*P*-value
Treatment	0.05	0.001
Genotype	0.26	0.001
Time	0.50	0.001
Treatment: genotype	0.03	0.001
Treatment: time	0.02	0.005
Genotype: time	0.05	0.001
Treatment: genotype: time	0.01	0.05
Residual	0.08	
Total	1	

The exudate variables include exudation rates per unit root surface area of total dissolved organic C, carbohydrates, amino acids, phenolics and carboxylates

Effect on the exudation variables is considered to be significant when *P*-value is below 0.05, *n* = 43

### Exudation rates of total C, carbohydrates, amino acids and phenolic compounds

**Fig. 2:**
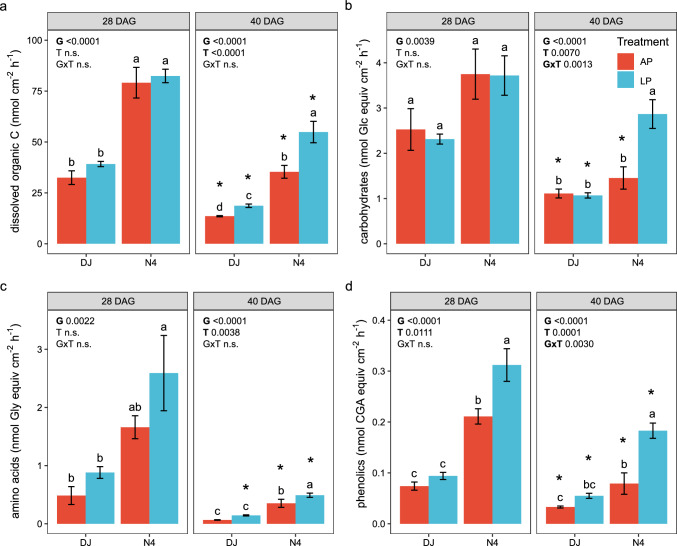
Root exudation rates per root surface area of rice genotypes (G) DJ123 (DJ) and Nerica4 (N4) in response to different P treatments (T) including adequate (AP) and low (LP) phosphorus supply at 28 and 40 days after germination (DAG). **a** Dissolved organic C (= total C). **b** Total soluble carbohydrates expressed in nmol Glc equivalents. **c** Total amino acids expressed in nmol Gly equivalents. **d** Total phenolic compounds expressed in CGA equivalents. Values represent means ± SE, *n* = 5–6. Different letters indicate significant differences within each time point (two-way ANOVA and LSD, *P* < 0.05). Asterisks indicate significant differences for each group between the two time points (two-sample unequal variance *t*-Test, *P* < 0.05). *Glc* glucose, *Gly* glycine, *CGA* chlorogenic acid, *equiv* equivalents, *AP* adequate phosphorus, *LP* low phosphorus, *G* genotype, *T* P treatment

Overall, exudation rates of dissolved organic C (total organic C), carbohydrates, amino acids and phenolics were higher at the earlier time point investigated (28 DAG), irrespective of genotype, P supply and exudate compound class investigated (Fig. 2a–d). Root exudation patterns differed significantly between the investigated genotypes and were partially affected by P treatment, with Nerica4 overall showing more pronounced changes under P deficiency, especially in carbohydrate, amino acids and phenolics exudation at the later time point (Fig. 2).

When averaged across both P treatments, P-inefficient Nerica4 had 2.3-fold and 3.1-fold higher exudation rates per root surface area of total C compared to P-efficient DJ123, at 28 DAG and 40 DAG, respectively. Exudation rates of individual exudate compound classes were also higher in P-inefficient Nerica4 with carbohydrate exudation rates being 1.5-fold and 2.1-fold, amino acid exudation rates 3.2-fold and 4.3-fold and phenolics exudation rates being 3.1-fold and 2.9-fold higher in Nerica4 when compared to P-efficient DJ123, at 28 DAG and 40 DAG, respectively (Fig. 2). Genotypic differences in exudation rates per entire plant were mostly not significant (Fig. S3).

P supply had no significant effect on genotype-specific total C exudation rates at 28 DAG, while at 40 DAG C exudation rates were 1.4-fold and 1.3-fold higher in LP compared to AP for DJ123 and Nerica4, respectively (Fig. 2a). Among the investigated exudate compound classes, at 28 DAG, amino acids (1.6-fold) and phenolics (1.5-fold) showed a significant increase in exudation rates in Nerica4 under low P supply while exudation rates of efficient DJ123 were only slightly enhanced and did not differ significantly when compared to adequate P supply (AP) (Fig. 2c, d). At 40 DAG, exudation rates of carbohydrates (1.8-fold), amino acids (1.2-fold) and phenolics (2.1-fold) were significantly higher in Nerica4 under low P supply when compared to the respective control. Efficient DJ123 showed a non-significant tendency of an elevated phenolic exudation rate at 40 DAG while all other investigated compound classes remained comparable to the control.

### Root exudation rates of carboxylates

**Fig. 3:**
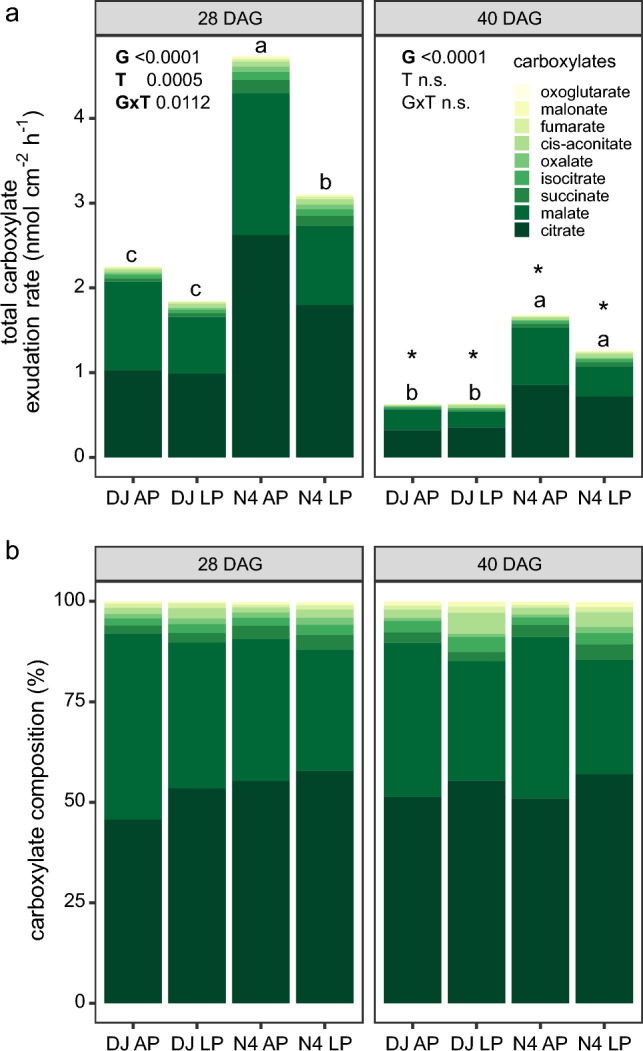
Root exudation rates of carboxylates per root surface area of rice genotypes (G) DJ123 (DJ) and Nerica4 (N4) in response to different P treatments (T) including adequate (AP) and low (LP) phosphorus supply at 28 and 40 days after germination (DAG). **a** Absolute exudation rates. Values represent means, *n* = 5–6. Different letters indicate significant differences across the different treatments and genotypes within each sampling event (two-way ANOVA and LSD, *P* < 0.05). Asterisks indicate significant differences for each group between the two time points (two-sample unequal variance *t*-Test, *P* < 0.05). **b** Relative contribution of individual organic anions (%) to total organic anions in root exudates. *AP* adequate phosphorus, *LP* low phosphorus, *DJ* DJ123, *N4* Nerica4

Irrespective of P supply and genotype, carboxylate exudation of the two investigated rice lines was dominated by citrate and malate and decreased with increasing plant age (Fig. 3). In contrast to our expectations, carboxylate exudation rates did not increase under P-limiting conditions and rather showed the opposite trend that was significant for Nerica4 at 28 DAG. When averaged across both P treatments, total carboxylate exudation rates were 1.9-fold and 2.3-fold higher in inefficient Nerica4 compared to efficient DJ123, at 28 and 40 DAG, respectively (Fig. 3a). Exudation rates of individual carboxylates are shown in the supplementary information (Fig. S2).

Across both sampling time points, the relative proportion of malate to the total amount of carboxylates exuded significantly decreased under P deficiency relative to control conditions (−9.3% and -8.3% for DJ123 and Nerica4, respectively, *P* < 0.01, *t*-test, Table S2), while the relative contribution of citrate significantly increased (+ 5.9% and + 4.1% for DJ123 and Nerica4, respectively, *P* < 0.05, *t*-test) (Fig. 3b). P deficiency effects on relative contribution of the other less abundant carboxylates were minor and not significant.

### Relative contribution of different exudate compound classes to total C exudation

**Fig. 4:**
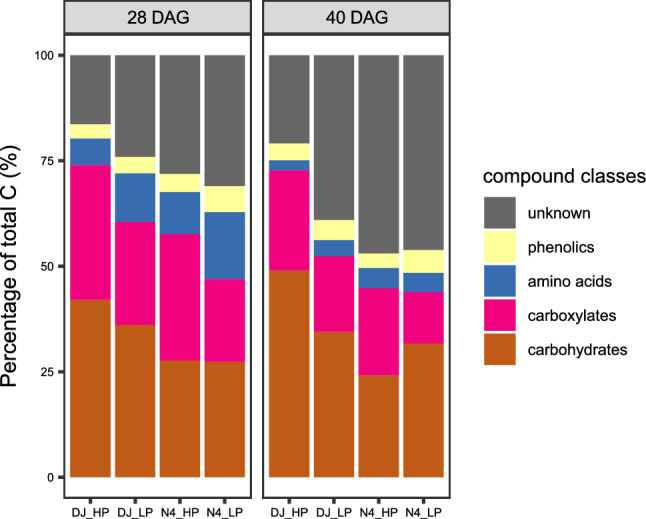
Estimated relative contribution of analyzed compound classes to total C exuded by rice genotypes DJ123 (DJ) and Nerica4 (N4) under adequate P supply (AP) and P deficiency (LP). *DAG* days after germination

The relative contribution of different exudate compound classes to total C exuded per unit root surface area (RSA) depended on genotype, P treatment and time of plant growth. Across all investigated treatments, carbohydrates were estimated to account for 24–49%, carboxylates for 12–35%, amino acids for 2–16% and phenolics for 3–6% of the total C exuded, leaving 17–47% of the exuded C unassigned (unknown compound class) (Fig. 4). Irrespective of genotype and P supply, the relative contribution of carboxylates and amino acids decreased with time, while the contribution of unknown exudate compounds to total C increased (*P* < 0.05, 3-way-ANOVA, Table S3). Relative contributions of carbohydrates and phenolics to total C were unaffected by time.

Irrespective of genotype and time point, proportion of carboxylates decreased significantly under P deficiency relative to the control, while the proportion of amino acids and phenolics increased (*P* < 0.05, 3-way ANOVA). Under P deficiency, the relative contribution of carboxylates to total C exuded decreased in DJ123 by −10.2% and −5.8% and in Nerica4 by −10.9% and −8.5% at 28 and 40 DAG, respectively. In contrast, relative proportion of amino acids increased in DJ123 by + 4.6% and + 1.4% at 28 and 40 DAG and in Nerica4 by + 5.7% only at 28 DAG under low P. Averaged across both time points, relative contribution of phenolics increased under P deficiency in comparison to the control by + 1.9% ± 0.2% (mean ± SE) for both genotypes. Only in DJ123 at 40 DAG, the relative contribution of carbohydrates and unknown compounds was affected by P treatment, with carbohydrates decreasing by -14.5% and unknown compounds increasing by + 18.1% under P deficiency relative to the control (*P* < 0.05, *t*-test, Table S4).

Comparing the two genotypes under P deficiency shows that DJ123 exuded a higher relative (but not absolute, see Fig. 2) proportion of carbohydrates (+ 8.5% and + 2.7% at 28 and 40 DAG, respectively) and carboxylates (+ 4.6% and + 5.7%) than Nerica4, whereas Nerica4 released higher proportions of unknown compounds (+ 7.1% and + 7.2%) and phenolics (+ 2.3% and + 0.67%) than DJ123 (*P* < 0.05, 3-way ANOVA).

## Discussion

### P deficiency triggers increased exudation rates in most investigated compound classes but not in carboxylates

This work aimed to reveal the response of root exudation rates to P limitation of two rice lines contrasting in P efficiency. Among the different root exudate compound classes investigated, carboxylates, especially the anions derived from tri- and di-carboxylic acids, are known to improve P solubilization by chelating Fe- or Al-oxide and releasing P (Hoffland et al. 1989; Oburger et al. 2011). Previous studies demonstrated an increased exudation of organic compounds in response to P deficiency for maize (Carvalhais et al. 2011; Ganie et al. 2015), rapeseed (*Brassica napus* L.) and rice (Hoffland et al. 1989, 2006), as well as for tomato (*Lycopersicon esculentum* L.), chickpea (*Cicer arietinum*) and white lupin (*Lupinus albu*s L.) (Neumann and Römheld 1999). Further studies investigating P uptake mechanisms in rice surmised that an enhanced exudation of carboxylates could play a role in an improved P mobilization from P-fixing soils (Mori et al. 2016; Wissuwa et al. 2020; Matsushima et al. 2021). Overall, we observed in most cases an increase in exudation rates of total C, carbohydrates, amino acids, and phenolics under P deficiency compared to adequate P supply (both genotypes and time points, Fig. 2). However, total carboxylate exudation rates showed the opposite trend with either no change or a significant decrease under P-limiting conditions (Fig. 3a). While our original hypothesis of higher overall exudation rates under low P (H1) was true for the majority of investigated rice exudate characteristics, we have to reject this hypothesis for carboxylates.

Among others, citrate and malate were the most abundant carboxylates exuded by the two rice lines tested (Fig. 3b). The quantities of citrate and malate released in our study were lower compared to P mining lupins (Shen et al. 2005; Wang et al. 2024), but were comparable to the quantities exuded by soil-grown rice (different genotypes) when converting exudation rates on a root dry weight basis (Aulakh et al. 2001; Bhattacharyya et al. 2013). For example, malate exudation rates ranged from 1.5 to 3.5 µmol per root dry weight g^−1^ h^−1^ across the three different rice studies. Regarding citrate exudation, contrasting reports were found for rice: an increased citrate exudation rate under P deficiency was reported in a semi-hydroponic experiment (Hoffland et al 2006), while no change or a decreased exudation rate of citrate was observed by Bhattacharyya et al. (2013) (soil-grown rice), in Tawaraya et al. (2013) (hydroponic) and in this study (semi-hydroponic). Comparison of the general carboxylate exudation patterns across different studies is difficult because different sets of carboxylates were analyzed as well as sampling setup (e.g., no bacteriostatic agent was used in the exudation sampling solution of the soil-grown plants) and duration differed. Based on available data from different rice genotypes and comparing the same developmental stage (before tillering), Hoffland et al. (2006) (semi-hydroponic) and Bhattacharyya et al. (2013) (soil) found increased total carboxylate exudation rates in rice under P deficiency. In contrast, this study observed no or decreased carboxylates (sum of analyzed) exudation rates under P limitation. Aulakh et al. (2001) who did not specifically target P deficiency found that carboxylates contributed around 39% to total C exuded in soil-grown rice plants of a similar growth stage, while we observed 12–35% carboxylate C, with a lower contribution to total C exuded from P starved plants. Taken together, this suggests that our results from semi-hydroponically grown plants represent reasonable insights into altered rice exudation patterns under P deficiency. Extrapolating from semi-hydroponic systems to the soil environment, the observed decrease in carboxylate exudation under P-limiting condition suggests a minor role of carboxylate exudation as relevant plant response to increase P mobilization. In other grass crops, such as wheat, P starvation was also reported to either have a negative or no effect on carboxylate exudation (Neumann and Römheld 1999; Pearse et al. 2006). This observation was related to the relatively low accumulation of citrate and malate in P limited wheat roots when compared to high exuding species such as white lupin or chickpea. Interestingly, the activity and the abundance of enzymes involved in citrate synthesis (PEPC) and degradation (aconitase) were both upregulated in wheat roots in response to low P, while PEPC activity was increased and aconitase activity was decreased in carboxylate exuding species (Neumann and Römheld 1999; Staudinger et al. 2022). Here, we only observed an increase of the relative contribution of citrate to the total amount of carboxylates exuded at the expense of malate, while absolute carboxylate exudation rates were either lower (28 DAG) under LP or comparable (40 DAG) to adequate P irrespective of genotype investigated (Fig. 3a). These results indicate that the citric acid (TCA) cycle in rice roots was reprogrammed, but that a substantial accumulation of citrate and malate in roots may have been limited by catabolizing enzymes.

In line with our results for rice, enhanced exudation of specific carbohydrates and amino acids in response to P limitation was also observed in maize (Carvalhais et al. 2011; Ganie et al. 2015). As carbohydrates (in the strict sense) present a net charge of zero, they are unlikely to contribute directly to nutrient mobilization in the rhizosphere. Amino acids can carry charges, depending on chemical structure, and the pH of the soil solution. However, a study on proteinogenic amino acids showed that these compounds have low P mobilizing capacity (Jones et al. 1994). Carbohydrates and amino acids are common respiratory substrates for soil microbes and present short residence times in soil (Jones and Murphy 2007). Therefore, they can contribute to plant nutrient availability through microbial feedback loops (Carvalhais et al. 2011). However, stress-induced increases in exudation rates of total carbohydrates, amino acids and phenolics were higher in P-inefficient Nerica4 and the higher exudation rates were associated with lower biomass production in Nerica4 relative to P-efficient DJ123, demonstrating that in this experimental setup (i.e., semi-hydroponic), potentially increased, exudation-induced plant–microbe feedback processes (mining) could not compensate for the smaller root biomass (exploration) of Nerica4. Whether the same results can be observed when plants are grown in natural soils, where more complex and diverse P sources and microbial communities can be expected, remains to be tested.

### P-efficient DJ123 had lower exudation rates per unit root surface area than P-inefficient Nerica4

In contrast to our original hypothesis (H2), we observed significantly lower exudation rates per unit root surface area of almost all investigated exudation parameters in P-efficient DJ123 compared to Nerica4, irrespective of P supply and sampling time point. Interestingly, total exudation per plant showed no or only minor differences between genotypes as the overall larger root system of DJ123 (with lower exudation rates) counterbalanced the higher exudation rate per unit root surface area in Nerica4 (with lower root biomass, Fig. S3). While the total exudate input per root system is relevant for soil C dynamics, normalizing root exudation data per unit root (per g dry weight or per cm^2^ root surface area, RSA) better allows addressing localized and spatially resolved rhizosphere processes like nutrient mobilization and we here focused on the latter.

Previous studies have shown that a larger root system enhances soil P foraging and subsequent P acquisition (Lynch and Brown 2001; Reichert et al. 2022). A larger root system, including longer roots (total root length) and larger root surface area of P-efficient genotype DJ123 compared to Nerica4 (Fig. S1), confirms the important role of root system size in P foraging and it explains the detected higher P uptake of DJ123 under P deficiency (Fig. 1c). Our biomass and root morphology data further confirm a previously reported overall slower root development in Nerica4 relative to P-efficient DJ123 (Wissuwa et al. 2020). Different root development stages between the two genotypes could have possibly driven the observed differences in exudation rates. Root exudation is unequally distributed across the root system with higher exudation being observed at root tips (Pausch and Kuzyakov 2011; Holz et al. 2017). Assuming that the proportion of strongly exuding root tips to the entire root system is larger in a less developed and smaller root system of Nerica4, this could further explain its overall higher exudation rates per unit root surface area.

Higher exudation rates of P-inefficient Nerica4 could also represent a way of discharging surplus C when an increasing amount of assimilated C allocated belowground cannot be used for growth due to limited P availability. To avoid accumulation of high concentrations of soluble C that would inhibit photosynthesis and lead to further oxidative stress in leaves (Prescott et al. 2020), exudation of this excess C could therefore simply be a mechanism of metabolic stress avoidance. This theory of increased exudation as a C surplus discharge mechanism when growth is limited due to environmental stress is supported by reports on exudation under drought stress where growth reduction is typically accompanied with an increase in (total C) exudation rates (Karst et al. 2017; Karlowsky et al. 2018; Preece et al. 2018). As Nerica4 generally produced less root and shoot biomass than DJ123 irrespective of P supply (Fig. 1a, b), it is likely that C allocated belowground was utilized for root biomass production in DJ123, but may have been discharged via exudation in the more growth-limited Nerica4. However, further evidence needs to be collected to support or reject the proposed mechanism of exudation being a C discharge strategy when belowground C allocation exceeds C demand for root growth.

### Rice exudation rates decreased with increasing plant development irrespective of P supply

As expected (H3), exudation rates of total C and measured compound classes were generally higher in younger rice plants (28 DAG) relative to plants grown for 12 more days (Figs. 2, 3). Our findings are supported by other studies including *Arabidopsis thaliana* grown hydroponically (Chaparro et al. 2013), maize grown under semi-hydroponic conditions (Gransee and Wittenmayer 2000) or in soil (Santangeli et al. 2024), and soil-grown rice (Aulakh et al. 2001). Returning to the theory of exudation as C discharge mechanism (Prescott et al. 2020), decreasing quantities of exuded C would imply changes in internal C partitioning over time. Ganther et al. (2022) reported decreasing allocation of freshly assimilated ^13^C to roots with increasing plant developmental stage in field grown maize which further supports that exudation is strongly influenced by the plant’s internal C allocation dynamics.

In addition to exudate quantity, we also investigated relative changes in exudate quality (i.e., estimated relative contribution (%) of different compound classes to total C exuded) with time (Fig. 4). Similar to previous reports on rice and maize (Aulakh et al. 2001; Santangeli et al. 2024), carbohydrates contributed most to total released C, while amino acids and phenolics represented a relatively small proportion (below 20%) of the total released C. Interestingly, the relative estimated proportion of unknown compounds, which were not assessed by our analytical approaches, increased under P deficiency relative to adequate P supply (Fig. 4), especially in DJ123. This suggests that P-efficient DJ123 might release a larger proportion of non-phenolic secondary metabolites to the growth substrate or soil when P is limited. Under P deficiency, increased exudation of many metabolites not targeted in this work (e.g., choline and polyamines) was found using non-targeted metabolomic analysis (Tawaraya et al. 2018). Therefore, involving non-targeted metabolomic analysis in further exudate studies of rice grown in soil or field (to also consider plant-soil-microbe feedback processes) could represent a promising approach when aiming to investigate the growing metabolite diversity under P deficiency (as suggested by our data) to further unravel the rhizosphere-related mechanisms of enhanced P acquisition suspected by previous studies (Mori et al. 2016; Matsushima et al. 2021).

## Conclusion

The contribution of root exudates to P acquisition in upland rice is not well understood. Our data confirms that the root system size plays a decisive role in higher P uptake of DJ123 under P deficiency. However, our results provide little evidence that changes in carboxylate exudation are a relevant response to mobilize P in the rice rhizosphere. In contrast to previous reports and expectations, total carboxylate exudation rates, which are considered the main P mobilizing exudates, mostly decreased under P-limiting conditions irrespective of genotype. However, total C, carbohydrates, amino acids, and phenolics exudation rates either increased or remained unchanged under P deficiency compared to adequate P supply. Interestingly, we observed that lower biomass production in P-inefficient Nerica4 was accompanied by higher exudation rates compared to P-efficient DJ123 that showed the opposite trend. This suggests that higher exudation rates may serve as a C surplus discharge mechanism under growth-limiting conditions. While we found no evidence of exudation significantly contributing to improved P uptake in our semi-hydroponic system, high C release into the rhizosphere could potentially trigger enhanced rhizobiome turnover and nutrient cycling leading to indirect P mobilization when rice plants are grown in soil. However, this remains to be tested. Despite focusing on only two genotypes, our results provide new insights into genotype-specific resource allocation under P limitation that warrant follow-up research including more genotypes.

## Supplementary Information

Below is the link to the electronic supplementary material.

Supplementary file1 (PDF 387 KB)

## Data Availability

Data will be available upon request.

## Abbreviations

AP: Adequate phosphorus
C: Carbon
DAG: Days after germination
LP: Low phosphorus
P: Phosphorus
