# Supplementary material for: Root exudation patterns of contrasting rice (Oryza sativa L.) lines in response to P limitation
Source: Planta. 2024 Oct 23;260(6):123. doi: 10.1007/s00425-024-04556-2 (PMC11499414; doi:10.1007/s00425-024-04556-2)
Supplement: Supplementary file 1 — Supplementary file1 (PDF 387 KB) [file 425_2024_4556_MOESM1_ESM.pdf]

Root exudation patterns of contrasting rice (*Oryza sativa* L.) lines in response to P limitation – supplementary information

Henning Schwalm[a*], Christiana Staudinger[a*, b], Mohammad-Reza Hajirezaei[c], Eva Mundschenk[d], Alireza Golestanifard[a], Maire Holz[d], Matthias Wissuwa[e], Eva Oburger[a]

[a] University of Natural Resources and Life Sciences, Vienna, Department of Forest and Soil Science, Institute of Soil Research, 3430, Tulln an der Donau, Austria

[b] University of Natural Resources and Life Sciences, Vienna, Department of Crop Sciences, Institute of Soil Research, 3430, Tulln an der Donau, Austria

[c] Leibniz Institute of Plant Genetics and Crop Plant Research, Department of Physiology and Cell Biology, Gartersleben, Germany

[d] Leibniz Centre for Agricultural Landscape Research (ZALF) e.V., Group of Isotope Biogeochemistry and Gas Fluxes, Müncheberg, Germany

[e] Crop, Livestock and Environment Division, Japan International Research Center for Agricultural Sciences, Tsukuba, Japan

*Henning Schwalm and Christiana Staudinger contributed equally

corresponding author: eva.oburger@boku.ac.at

Root morphological traits

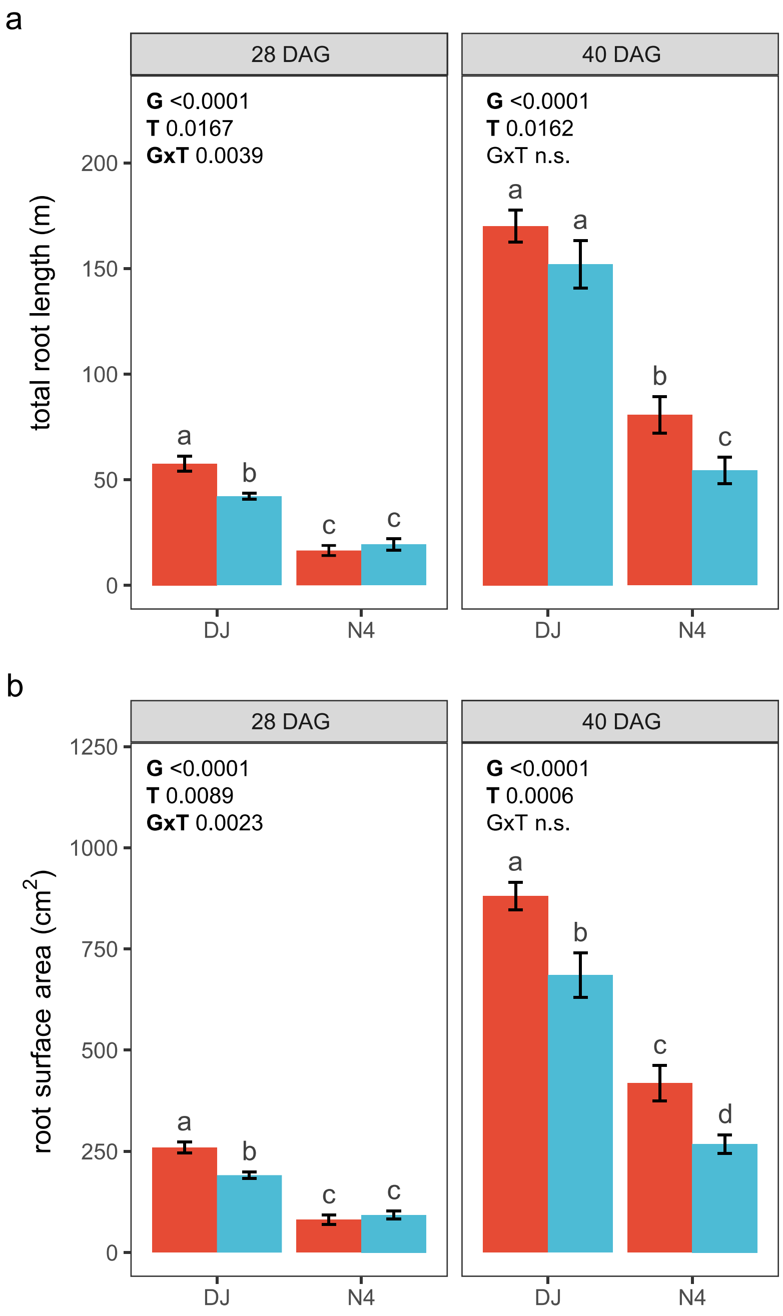

Fig. S1 Root morphological traits of rice genotypes (G) DJ123 (DJ) and Nerica4 (N4) in response to different P treatments (T) including adequate (AP) and low (LP) phosphorus supply at 28 and 40 DAG. Morphological traits comprise total root length (**a**) and root surface area (**b**). Values represent means ± SE, *n* = 5-6. Different letters indicate significant differences across different P treatments and genotypes within each sampling event (two-way ANOVA and LSD, $P < 0.05$). DAG = days after germination, AP = adequate phosphorus, LP = low phosphorus

Individual carboxylate exudation rates per root surface area

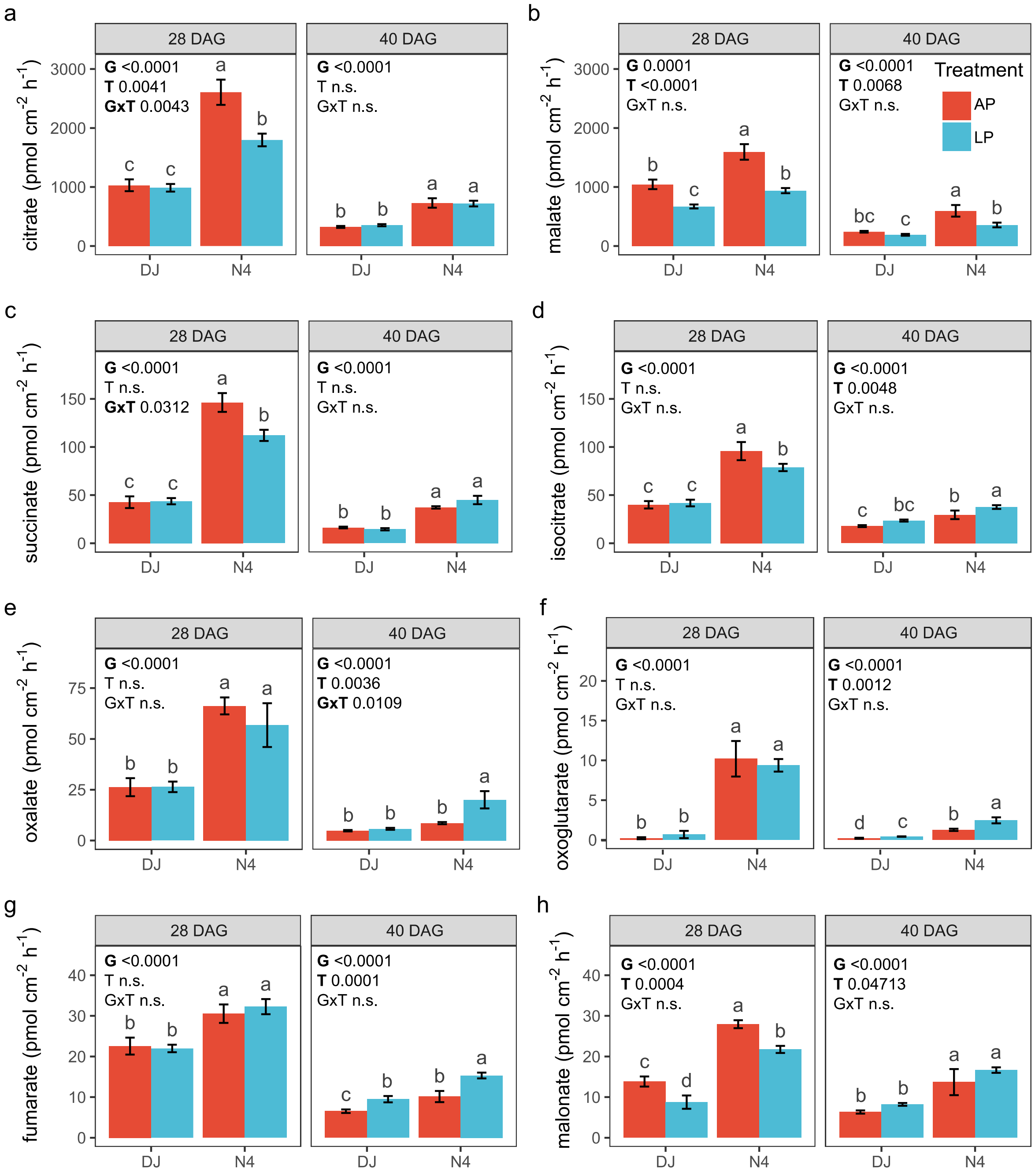

Fig. S2 Root exudation rates of carboxylates in pmol per root surface area of rice genotypes (G) DJ123 (DJ) and Nerica4 (N4) in response to different P treatments (T) including adequate (AP) and low (LP) phosphorus supply at 28 and 40 days after germination (DAG). **a** Citrate. **b** Malate. **c** Succinate. **d** Isocitrate. **e** Oxalate. **f** Oxoglutarate. **g** Fumarate. **h** Malonate. Values represent means ± SE, n = 5 - 6. Different letters indicate significant differences within each time point (two-way ANOVA and LSD, $P < 0.05$). AP = adequate phosphorus, LP = low phosphorus

Exudation rates of total C, carbohydrates, amino acids and phenolics per plant

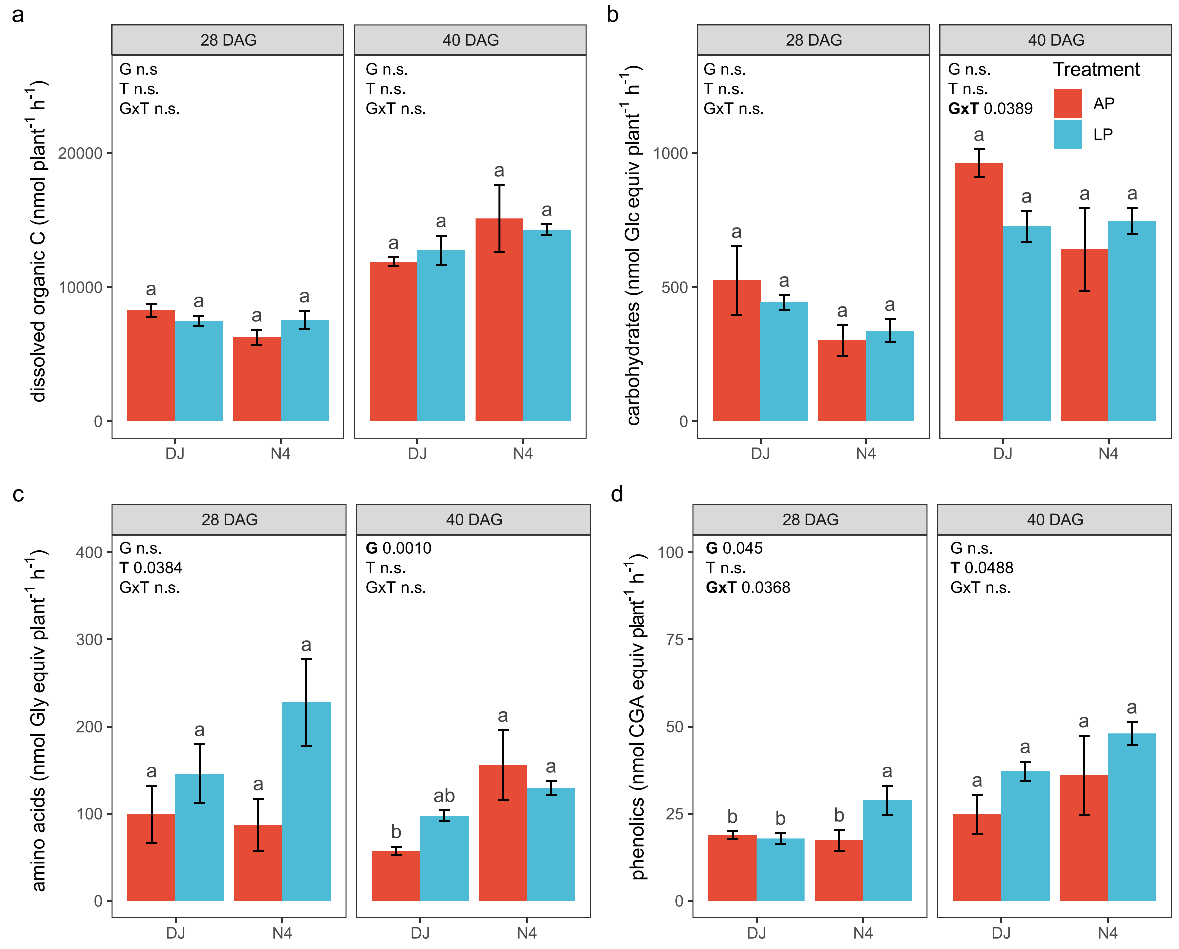

Fig. S3 Root exudation rates per plant of rice genotypes (G) DJ123 (DJ) and Nerica4 (N4) in response to different P treatments (T) including adequate (AP) and low (LP) phosphorus supply at 28 and 40 days after germination (DAG). (**a**) dissolved organic C (= total C), (**b**) total soluble carbohydrates expressed in nmol Glc equivalents, (**c**) total amino acids expressed in nmol Gly equivalents and (**d**) total phenolic compounds expressed in CGA equivalents. Values represent means ± SE, n = 5 - 6. Different letters indicate significant differences within each time point (two-way ANOVA and LSD, $P < 0.05$). Glc = glucose, Gly = glycine, CGA = chlorogenic acid, equiv = equivalents, AP = adequate phosphorus, LP = low phosphorus, G = genotype, T = P treatment

Two-sample unequal variance t-test

1. Time effect on exudation of total C and targeted compound classes

Pairwise comparison between the two time points (i.e., 28 and 40 days after germination (DAG)) to assess the effect of time on exudation rates of total C, carbohydrates, amino acids, phenolics and carboxylates is shown as p-values in the following table. Time effect on exudation rate is significant when *P*-value is below 0.05 (shown as asterisks in Fig. 2 in chapter 3.3 and in Fig. 3a in chapter 3.4).

Table S1 *P*-value results derived from pairwise comparison between two sampling time points (28 and 40 days after germination (DAG)) to assess time effect on exudation rates per root surface area of dissolved organic carbon (DOC), carbohydrates, amino acids, phenolics and carboxylates (two-sample unequal variance t-test, $P < 0.05$). Example of pairwise comparison between time points: DJ AP at 28 DAG vs DJ AP at 40 DAG. DJ = genotype DJ123, N4 = genotype Nerica4, AP = adequate phosphorus treatment, LP = low phosphorus treatment. p-values in red indicate significant differences in exudation rate between two time points (*n* = 5-6)

28 vs 40 DAG	DOC	carbohydrates	amino acids	phenolics	carboxylates
**DJ AP**	0.0024	0.0357	0.0523	0.0030	0.0003
**DJ LP**	< 0.0001	< 0.0001	0.0018	0.0014	< 0.0001
**N4 AP**	0.0165	0.0055	0.0009	0.0007	0.0001
**N4 LP**	0.0009	0.1039	0.0225	0.0070	< 0.0001

2. Treatment effect on the relative contribution of carboxylates

Pairwise comparisons between P treatments to assess changes in the relative contribution of different carboxylates to the total carboxylates (Table S2). Treatment effect on the relative contribution of carboxylates is significant when *P*-value is below 0.05 (shown in text of chapters 3.4).

Table S2 p-value results derived from pairwise comparison between two phosphorus treatments (AP vs LP) for both genotypes (averaged across time points) to assess P treatment effect on the contribution of individual carboxylates to total carboxylate exudation (two-sample unequal variance t-test, $P < 0.05$). DJ = genotype DJ123, N4 = genotype Nerica4, AP = adequate phosphorus treatment, LP = low phosphorus treatment. *P*-values in red indicate significant differences in the contribution of an individual carboxylate to total carboxylate exudation (*n* = 5-6)

AP vs LP	DJ	N4
**% Oxoglutarate**	0.0593	0.0260
**% Malonate**	0.5730	0.1286
**% Fumarate**	0.0002	< 0.0001
**% Cis-aconitate**	0.0004	0.0086
**% Oxalate**	0.3572	0.0178
**% Isocitrate**	0.0393	< 0.0001
**% Succinate**	0.6110	0.0209
**% Malate**	0.0001	0.0008
**% Citrate**	0.0002	0.0202

3. P-Treatment, genotype and time effect on the relative contribution of different compound classes to total C exuded

Three-way ANOVA (Analysis of Variance) to determine the effect of three independent factors (P-treatment, genotype, time point) on the relative contribution of different exudate compound classes to the total C exuded per unit root surface area (Table S3). Effect on the relative contribution of exudate compounds is significant when *P*-value is below 0.05 (shown in chapter 3.5).

Table S3 *P*-value results derived from three-way ANOVA to assess effect of phosphorus treatment, genotype and time point on the relative contribution of different exudate compound classes to the total C exuded per unit root surface area. Data for relative contribution of amino acids were log-transformed to approximate normality and homoscedasticity assumptions. *P*-values in red indicate significant differences in the contribution of an individual compound classes to total carbon exudation (n = 5-6)

	% carbohydrates	% amino acids	% phenolics	% carboxylates	% unknown
**treatment**	0.0776	0.0125	0.0015	< 0.0001	0.1259
**genotype**	0.0001	0.0049	0.0286	< 0.0001	0.0039
**time**	0.8215	< 0.0001	0.7489	< 0.0001	0.0037

Although the three-way ANOVA showed no significant P treatment effect on the contribution of carbohydrates and unknown compounds to total C exuded, an additional pairwise comparisons between P treatments for each genotype was separately done to identify possible P treatment effects on those compounds when only focusing on one genotype. The additional t-test revealed no P treatment effect in Nerica4, but significant differences between P deficiency (LP) and the control (AP) in the contribution of carbohydrates and unknown compounds in DJ123 at 40 DAG (Table S4).

Table S4 *P*-value results derived from pairwise comparison between two phosphorus treatments (HP vs LP) for both genotypes and both time points separately to assess P treatment effect on the contribution of carbohydrates and unknown compounds to total carbon exudation (two-sample unequal variance t-test, $P < 0.05$). DJ = genotype DJ123, N4 = genotype Nerica4, AP = adequate phosphorus treatment, LP = low phosphorus treatment. *P*-values in red indicate significant differences in the contribution of an individual carboxylate to total carboxylate exudation (n = 5-6)

LP vs AP in DJ	28 DAG	40 DAG
**% unknown**	0.6428	0.0230
**% carbohydrates**	0.1845	0.0080
**LP vs AP in N4**	**28 DAG**	**40 DAG**
**% unknown**	0.7896	0.9045
**% carbohydrates**	0.8314	0.1004
